# Horizontal transmission of streptococcus mutans in schoolchildren

**DOI:** 10.4317/medoral.17592

**Published:** 2011-12-06

**Authors:** Pilar Baca, Ana M. Castillo, Maria J. Liébana, Francisca Castillo, Antonio Martín-Platero, José Liébana

**Affiliations:** 1 Department of Preventive Dentistry. University of Granada, Spain; 2 Department of Microbiology. University of Granada, Spain; 3 ….

## Abstract

Objetive: The aim of this study was to analyze possible horizontal transmission patterns of S. mutans among 6-7-yr-old schoolchildren from the same class, identifying genotypes and their diversity and relationship with caries disease status. Study Design: Caries indexes and saliva mutans streptococci and lactobacilli counts were recorded in 42 schoolchildren. Mutans streptococci colonies were identified by means of biochemical tests and all S. mutans strains were genotyped by arbitrarily primed polymerase chain reaction. A child was considered free of S. mutans when it could not be isolated in 3 samples at 1-week intervals. Results: S. mutans was isolated in 30 schoolchildren: 20 having one genotype and 10 two genotypes. Higher mutans streptococci and caries index values were found in those with two genotypes. Five genotypes were isolated in more than 1 schoolchild and one of these was isolated in 3 schoolchildren. Our results suggest that horizontal transmission may take place. Conclusion: Schoolchildren aged 6-7 yrs may be the source of mutual transmission of S. mutans.

** Key words:**Streptococcus mutans, Horizontal transmission, AP-PCR, genotyping

## Introduction

Mutants streptococci (MS) are microorganisms associated with the development of caries, and Streptococcus mutants is the most frequently isolated member of this group in humans (1). Colonization of this bacterium is related to tooth eruption. It has been suggested that organisms are acquired during a “window of infectivity” at 19-31 months of age (2). A second “window” has also been proposed at 6-12 yrs of age, coinciding with eruption of the permanent dentition (3).

Phenotyping and genotyping studies of intrafamily clonal distribution suggested that the mother is the major primary source of infection for children with MS strains (4.) However, detection in children of genotypes not found in their mothers or relatives indicates the existence of other sources of transmission. For review see (5) and (6). Some evidence has been reported of horizontal transmission among children at the same nursery school (7,8), although results have been controversial (9) and studies always confined to nursery school groups. Further studies of this phenomenon are warranted among older schoolchildren.

Information is needed on the different sources of infection and colonization by this microorganism in children in order to develop preventive strategies against dental caries (10,11). This paper presents a cross-sectional evaluation of S. mutants genotypes isolated in saliva of 6-7 year-old children in the same class and analyzes possible patterns of horizontal transmission among the children, the genotypic diversity in each schoolchild, and its relationship with caries disease status.

## Material and Methods

Study population 

The study was approved by the Ethics Committee of the University Hospital, Granada (Spain). One school was randomly selected in the Northern district of the city. The school had two first-year classes of 25 pupils each whose parents gave written informed consent, with 42 finally participating. None had chronic diseases or had received antibiotic treatment, and none had used any mouthwash in the previous 4 weeks. There was no school-based preventive dental program. The drinking water in the area had 0.07 ppm F ion. The children attended school five days a week, 6 h per day.

Examination and bacterial sampling 

Caries history in permanent and deciduous teeth was recorded using World Health Organization methodology and criteria (12). A single calibrated dentist carried out all examinations. A second dentist participated to test inter-examiner reliability. Intra -and inter-examiner diagnostic agreement was analyzed in ten children with an interval of 5-7 days. Reliability was analyzed by using the intraclass correlation coefficient, obtaining values >0.80.

Stimulated (chewing 1 g of sterile paraffin for 5 min) whole saliva samples were obtained from each child at around 10 am and immediately delivered in ice to the laboratory. Salivary samples were dispersed on a magnetic agitator for 20 s and tenfold diluted in 0.05M phosphate buffer with 0.4% (w/v) KCl (pH 7.1). Aliquots of 0.1 ml were plated on MSB agar (13) (Difco; Detroit, MI) for enumeration of MS and on Rogosa SL medium (14) (Difco) for lactobacilli (LB). Total colony-forming units (CFU) with characteristic morphology of MS or LB were counted after incubating at 37ºC: 48 h under 10% CO2 atmosphere. Up to three repeat samplings were performed at 1 week intervals if MS could not be recovered. After three repeats with no detection, children were considered MS-negative.

Microbiological processing

All possible MS colony morphotypes were selected, and 5-20 isolates (when available) were recovered from each child, with a mean (standard deviation) of 8.42 (5.53) isolates. Sample colonies were transferred to 2 ml of BHI and grown for 24 h. Identification of MS was confirmed by biochemical tests (15,16). Isolates were then pure cultured into 50% glycerol and stored at -70ºC for later DNA extraction. All isolates identified as S. mutants were genotyped by AP-PCR.

Preparation of DNA and Polymerase Chain Reaction (AP-PCR).

An aliquot of glycerol-preserved strains was injected into Wilkins Chalgren Broth (Panreac Quimica SA, Barcelona, Spain) and incubated overnight. The DNA extraction used a Wizard® Genomic DNA Purificationn Kit, following manufacturer`s instructions (Promega Corporation, Madison, WI). Extracts were kept at -20ºC until use. The amplification was performed following previously published criteria (17). Briefly, for a total volume of 50 µl, the mixture for each reaction contained 200 µM of each of the deoxynucleotides dATP, dCTP, dGTP and dTTP (Promega Corporation), 3.5 mM of Cl2Mg (Panreac Quimica SA), 1.25 units of Taq-polymerase (Promega Corporation); 5µl of 10x buffer (20 mM Tris-HCl pH 8, 100 mM KCl, 0.1 mM of EDTA, 1mM DTT, 50% glycerol, 0.5% Tween®20, and 0.5% Nonidet®-P40); 50 pmol of primer OPA-02 (5`-TGCCGAGCTG-3`) (Tib Molbiol, Berlín, Germany) and 20 ng of DNA extract (4200 UV-VIS4418 Zuzi scanning spectrophotometer, Beijing, China). The reaction was carried out in an Eppendorf AG Mastercycler® thermocycler (Eppendorf, Hamburg, Germany), heating at 94ºC for 2 min followed by 45 cycles of 94ºC for 30 s, 36ºC for 30 s, and 72ºC for 1 min. The final extension to complete the amplification was 5 min at 72ºC.

PCR product was analyzed by electrophoresis on 1.5% agarose gel prepared with 10x TBE (Promega Corporation) and 1µg/ml of ethidium bromide (Promega Corporation). DNA molecular weight marker XIV (1 721 933 Roche Diagnostic GMBH, Roche Applied Science, Mannheim, Germany) and the same time and voltage conditions (4 hrs, 100 volts) were always used for maximum uniformity. Bands were visualized on an ultraviolet transilluminator (Vilber Lourmat, Marne la Vallée Cedex, France). Images were photographed using a digital camera (Nikon Coolpix 4500, Tokyo, Japan) and stored in Tagged Image File Format.

AP-PCR Typing of S. mutants

AP-PCR showed a good reproducibility; isolates typed at least three times with AP-PCR always showed identical profiles. AP-PCR banding patterns were analyzed by side-by-side visual comparison of gels. All strains from the same child were run together on a single gel to ensure good conditions for comparison. Isolates were rerun on the same gel when results of the comparison of different gels were doubtful and when similar DNA fingerprints were seen on different gels. Bands were scored visually as present or absent by means of an image analysis program (Adobe Photo shop), marking bands with the cursor. Each image was scored by three independent observers. Fingerprints were considered similar when major bands were identical and minor bands showed ≤ 2 differences (18). Images were also analyzed using Fingerprinting II informatix TM Software (BioRad Laboratories, Inc., CAL) although, as previously reported (19), manual scoring appeared more reliable.

Data analysis

MS and LB counts in CFU/ml of saliva were transformed by log10 (CFU+1) before statistical analysis. After confirming the Gaussian distribution of these variables, a Student’s t-test was used to compare mean MS and LB levels between children with one and two genotypes. Caries index values ( Table 1) did not follow a normal distribution and were compared between groups using the Kolmogorov-Smirnov test. In all tests, p < 0.05 was considered statistically significant.

**Table 1:**
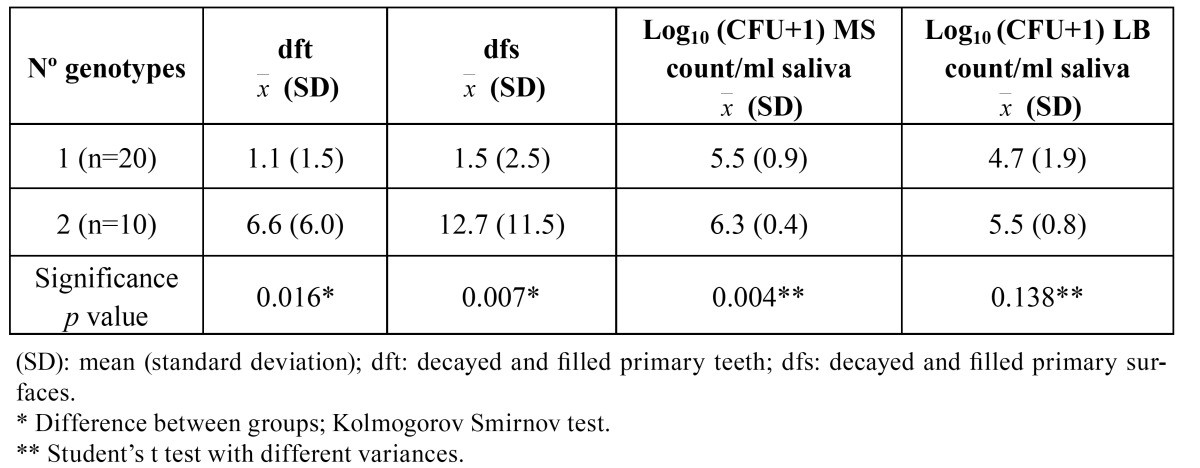
Caries index values and mutans streptococci (MS) and lactobacilli (LB) levels in saliva in children with 1 and 2 S. mutans genotypes.

## Results

Mean (SD) age of children was 6.8 (0.23) yrs, 57% were male, and decayed and filled primary teeth (dft) and surfaces (dfs) index values were 2.14 ( 3.95) and 3.79 (7.61), respectively. MS and LB counts/ml saliva were 4.13 (2.74) and 4.41 (2.07), respectively. A mean of 3.76 (0.48) 6-year molars were erupted. Twenty of the children (47.61%) were caries-free in primary dentition, although one of these showed the only caries detected in permanent teeth.

S. mutants was detected on MSB plates corresponding to 30 children (71.42%), of whom 6 were also colonized with S. sobrinus, which was never isolated alone. There were no significant differences in caries index values or MS or LB levels between children with S. mutants alone and children with both S. mutants and S. sobrinus. Among the 12 children with no S. mutants isolated in saliva, 11 had no caries and 1 had two surfaces with caries in one primary tooth.

In the whole series, 354 colonies were isolated. Biochemical identification of isolates showed, as expected, a more frequent isolation of S. mutants (63.27 %) than of S. sobrinus (4.51%), Streptococcus criceti (2.5%), or Streptococcus ratti (0.8%). A large number of isolates (29.37%) did not belong to the MS group.

A total of 224 S. mutants isolates were obtained in saliva samples from 30 children, a mean (SD) of 7.47 (3.88); 20 children harbored one genotype and 10 two genotypes; no children harbored three or more genotypes.

The ( Table 1) shows mean dft and dfs index values and MS and LB (log10-transformed) levels as a function of number of genotypes. A significant relationship was found among number of genotypes, caries index value, and MS count. Both dft and dfs caries index values were significantly associated with saliva MS levels but not with LB levels (results not shown).

**Figure 1:**
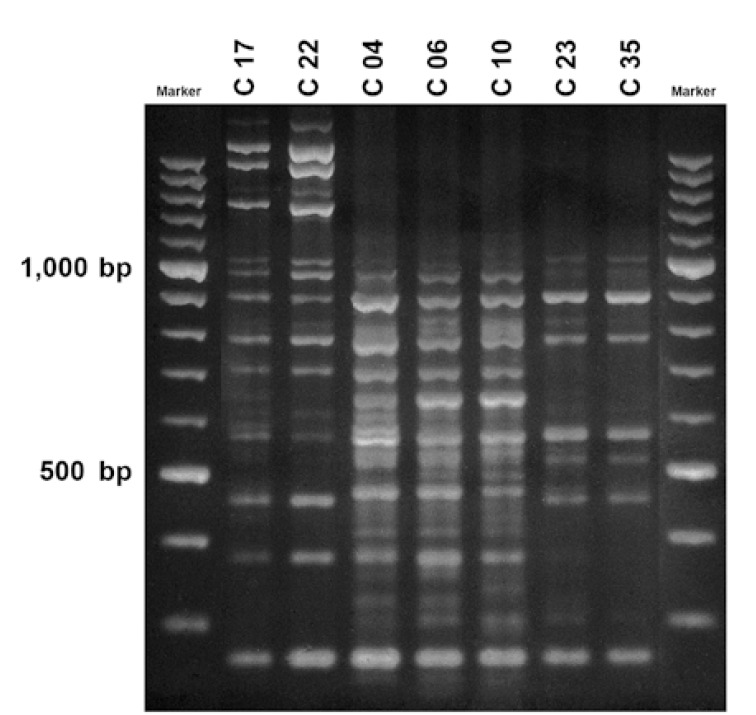
AP-PCR fingerprint of seven schoolchildren. Three genotypes of S. mutans are shown: one isolated in children 17 and 22, another in children 04, 06, and 10, and the third in children 23 and 35.

Among the 30 S. mutants-positive schoolchildren, 11 shared a genotype with at least one other child. Thus, 34 different genotypes were identified: 1 isolated in three schoolchildren, 4 in two, and 29 in each of twenty-nine schoolchildren (Fig.1). Out of these 11 children, five also showed a genotype other than the shared one. All of the shared genotypes except one were found in children from the same class.

## Discussion

Identification of the source of MS transmission is essential for developing preventive strategies for dental caries. Several studies have demonstrated the presence of matching genotypes of S. mutants in mother-child pairs, suggesting vertical transmission (5) and the oral health care of pregnant women may cause sustained and long-term improvement of the oral health of children (10,11). However, horizontal transmission has been less studied and results have been contradictory (7,9,20,21). The present finding of matching genotypes in schoolchildren from the same class supports the theory of horizontal transmission.

The prevalence of S. mutants-positivity in this group of 6-7-yr-old children was 71.42%, similar to results in younger nursery school children (22-24) as well as in other 6-7-yr-olds. A longitudinal study of children whose mothers were colonized by S. mutants and S. sobrinus reported that only 10 out of 15 children acquired MS during the first 7 years of life (25). These data support the proposition of a second, later “window” of colonization.

Despite only selecting colonies with characteristic MS morphology, a very high percentage of colonies isolated on MSB agar were not identified as MS. Hence, MSB medium is not exclusively selective for MS, as noted by other authors (26,27). Our results verify, in agreement with previous studies (17,28), that OPA-02 is a useful primer for typing strains of S. mutants and producing reproducible amplicons.

These children showed a low genotype biodiversity, with only one genotype isolated in most of them. Although reports of the number of genotypes per individual vary widely, it is not unusual to find a higher prevalence of one genotype per individual (7,29,30). Discrepancies among study results may derive from the use of distinct detection methods or from differences in the ethnic background of populations (31). Although this study used saliva sampling, has been previously reported that it was equally as effective as plaque sampling (32).

Children with two genotypes showed higher caries index values and higher SM counts versus those with one genotype ( Table 1). Studies comparing genotypic diversity with MS levels or caries prevalence have produced contradictory results. Thus, Mattos Graner et al. (7) found no relationship between caries and genotype diversity. However, our finding supports a previous report of a higher number of genotypes in caries-active versus caries-free subjects (33). It is possible that a higher level of colonization and greater genotype diversity may result from a more cariogenic diet, with all factors increasing caries risk.

The present study showed that 17.64% of all different genotypes found (n=34) could be isolated in more than one schoolchild, with one genotype even isolated in 3 schoolchildren. This result is unlikely to be a chance finding, especially given that only one shared genotype was isolated in children from different classes. Reports on mother-child vertical transmission, which range from 24% (34) to 81% (9), imply the identification of numerous genotypes with an unknown source of transmission, which may represent indirect evidence of horizontal transmission. Genotypes were not investigated in mothers, other relatives, or teachers in the present study, but matching genotypes among children were found. This finding is consistent with the observation by Lindquist and Emilson (25) that S. mutants may be acquired from external sources when children increase their social contacts outside the family.

Some characteristics of the study population may have influenced horizontal transmission. Thus, S. mutants was not detected in a relatively high percentage of the present children, who may undergo a late colonization after longer contact with their schoolmates. Moreover, children who are already colonized can acquire new strains, since the microflora of children is not completely stable (25). In this age group, eruption of the 6-year molar may have favored colonization (35). It should also be borne in mind that these children spend around 30 h per week together in the classroom. Finally, bacterial factors may have played a role, given the greater virulence of some strains, enhancing their colonization and survival (36).

Since colonization of early acquired strains has been reported to persist into young adulthood (37), there is a need for school-based programs (e.g., chlorhexidine varnish treatment) to prevent, delay, or reduce the horizontal transmission identified in this study.

Within the limitations of this study and taking into account the methodology used, our results suggest that horizontal transmission may take place. Schoolchildren aged 6-7 yrs may be the source of mutual transmission of S. mutants. Further research is required to elucidate transmission routes in order to optimize anti-caries measures.
